# Impact of energy consumption on environment sustainability in upholding ESG practices in Malaysia: Evidence from electricity supply company

**DOI:** 10.1371/journal.pone.0327744

**Published:** 2025-08-01

**Authors:** Muhummad Khairul Islam, Muhammad Nazmul Hoque, Kazi Musa, Nisaa Husnina Binti Zulkifli

**Affiliations:** 1 Universiti Tenaga Nasional, Kajang, Malaysia; 2 Faculty of Accountancy, Universiti Teknologi MARA Cawangan Selangor, Kampus Puncak Alam, Selangor, Malaysia; 3 Accounting Research Institute, Universiti Teknologi MARA, Selangor, Malaysia; Universiti Teknologi Petronas: Universiti Teknologi PETRONAS, MALAYSIA

## Abstract

This study aims to examine the impact of energy consumption, i.e., coal and gas, on environmental sustainability by utilizing CO2 emissions as a proxy to emphasize Environmental, Social, and Governance (ESG) practices in the electricity supply company of Malaysia. To do so, we collect time series data from Tenaga Nasional Berhad (TNB) Sustainability Reports spanning 2016–2021. Since the data is short time series data, this study deploys the Prais-Winsten (AR1) regression technique, which is able to produce robust estimates from the short data. Besides, this method is also able to overcome the issues of heteroscedasticity and autocorrelation issues in the dataset. Additionally, we also employ the Ordinary Least Square (OLS) regression method to check the robustness of our estimation. The findings of the study reveal that energy consumption, i.e., coal and gas, strongly affects the environmental sustainability channel through CO2 emissions. The result indicates that the electricity supply company of Malaysia still aggravates the environmental degradation using coal and gas in energy production, while the study also shows from the long-run trends that the sector has considerably decreased the CO2 emissions in recent years by 0.60%. Since the emission is still significant, this study emphasizes the need for a cautious approach to energy sources in order to reduce environmental effects. The study suggests that policymakers should review the existing energy use and CO2 emissions policy to strengthen environmental sustainability and ESG standards in Malaysia’s electricity generation industry, reduce dependency on fossil fuels, and hasten the adoption of renewable energy sources.

## 1. Introduction

In today’s global landscape, the intersection of energy consumption and environmental sustainability has become paramount, especially within the realm of Environmental, Social, and Governance (ESG) practices [1]. This nexus is particularly significant in Malaysia, where the electricity supply sector stands as a pivotal player in the nation’s economic development and societal progress [2]. The current article aims to delve into the impact of energy consumption on environmental sustainability and how it resonates with ESG practices within Malaysia’s Electricity Supply Company.

Malaysia’s Electricity Supply Company operates within a dynamic ecosystem where the pursuit of environmental sustainability is closely connected with ESG principles [3]. As the nation embraces sustainable development goals, the electricity production sector faces mounting pressure to mitigate its environmental footprint while meeting the burgeoning energy demands of a growing economy. The incorporation of ESG practices serves as a guiding framework for companies to navigate these challenges while aligning with global sustainability agendas [4].

Within the operations of Malaysia’s Electricity Supply Company, various energy sources are harnessed to power homes, businesses, and industries across the nation [5]. From traditional fossil fuels like coal and natural gas to renewable sources such as hydroelectric, solar, and wind energy, the energy mix is multifaceted. Each energy source brings its own set of opportunities and challenges concerning environmental impact, resource availability, and technological advancement [6]. However, the high dependence on coal and gas in the Malaysian electricity production sector might be one of the strong barriers to reaching environmental sustainability in this country. This overdependence of environmentally vulnerable energy sources in producing electricity is the main motivation to conduct this study.

**Fig 1:**
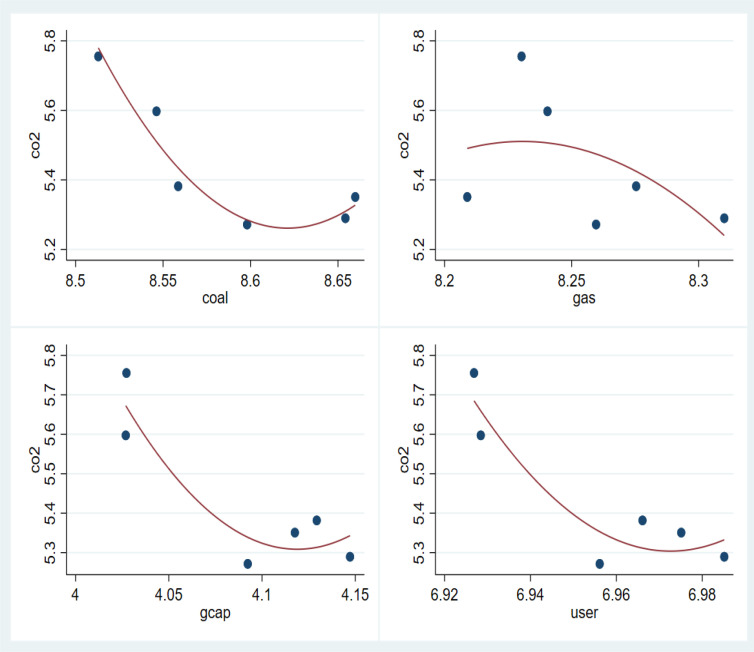
Long Trends of the Dependent and Independent Variables. Source: Author's compilation.

The utilization of various energy sources within Malaysia’s Electricity Supply Company underscores the intricate balance between energy generation and environmental sustainability [7]. Fig 1 below shows the long-term trends of CO2 emissions from coal and gas in the electricity production sector of Malaysia. The vertical axis represents different levels of CO2 emissions, while the X-axis displays the other variables: coal, gas, gcap, and user and their levels.

While conventional sources like coal and natural gas offer reliability and affordability, they also contribute significantly to carbon emissions and environmental degradation. Conversely, renewable energy options promise cleaner alternatives but often require substantial investments in infrastructure and technology [8]. Fig 1 demonstrates that coal and gas contribute significantly to CO2 emissions, but in recent years, the emissions have risen considerably in the electricity production sector of Malaysia. The figure also presents data on the control variables. Additionally, the following figure highlights a significant rise in coal and natural gas usage as a source of carbon emissions within the electricity production sector.

Through the lens of Environmental, Social, and Governance (ESG) practices, Malaysia’s electricity supply companies are increasingly striving to balance the often competing goals of energy security, economic viability, and environmental stewardship [9]. By embedding environmental considerations into corporate strategies, such as investing in renewable energy, enhancing energy efficiency, and adopting innovative low carbon technologies, these companies aim to reduce their ecological footprint while advancing long term sustainability objectives [10].

**Fig 2:**
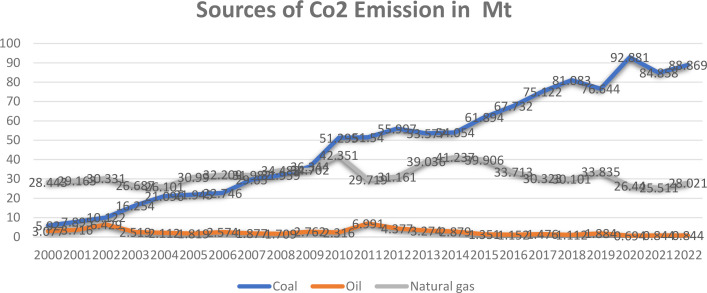
Evolution of emissions from power generation by source in Malaysia since 2000. Source: International Energy Agency (2025).

Despite these progressive steps, the current energy mix remains heavily reliant on fossil fuels. In 2022, coal accounted for approximately 47 percent and natural gas for 34 percent of total electricity production. Moreover, the consumption of both coal and natural gas in power generation has increased markedly over the past two decades, highlighting a growing dependence on carbon intensive energy sources. This trend (Fig 2) presents a critical challenge to Malaysia’s ambition of achieving net zero emissions by 2050, as outlined in national energy and climate policies. It is worth mentinaing that, as of 2022, about 80 percent of electricity came from coal (46.8 percent) and natural gas (34.3 percent), with hydro and other renewables contributing just 18 percent [11].

Given this context, it is essential to focus empirical research on electricity supply companies to examine the impact of energy consumption on environmental sustainability. A detailed understanding of this relationship can offer evidence based insights for policy formulation, particularly in promoting ESG aligned initiatives across the energy sector. By identifying the specific ways in which energy consumption patterns influence carbon emissions, such research can support the design of targeted interventions and regulatory frameworks needed to steer the country toward a greener and more sustainable future.

Businesses today are increasingly expected to operate responsibly, and Tenaga Nasional Berhad (TNB), one of Malaysia’s primary electricity producers, demonstrates this commitment by prioritising environmental sustainability. In 2023, TNB invested RM1.48 million in initiatives promoting eco friendly practices, biodiversity conservation, and community engagement. Its “My Brighter Green” programme, along with the Sustainability Roadmap 2050, underscores the company’s goal to achieve net zero carbon emissions by 2050 [12]. These efforts are also aligned with the government’s Greening Malaysia Programme, which targets the planting of 100 million trees by 2025.

**Fig 3:**
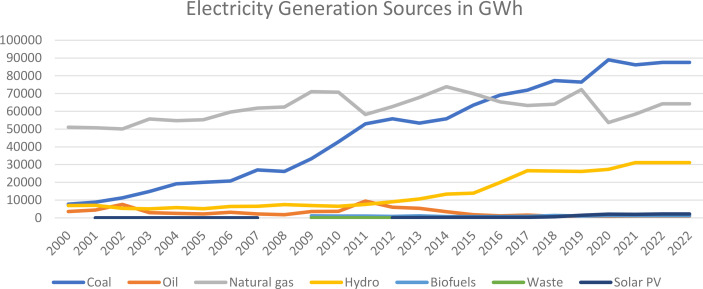
Evolution of electricity generation sources in Malaysia since 2000. Source: International Energy Agency (2025).

Despite progress in incorporating ESG principles, several research gaps remain within Malaysia’s electricity production sector. First, there is a lack of comprehensive empirical analysis of environmental sustainability, particularly in relation to CO_2_ emissions from coal and gas consumption, two major sources of energy in this sector. As illustrated in Figs 2 and 3, both energy usage and associated carbon emissions have significantly increased since 2000. However, most existing studies rely heavily on theoretical discussions of ESG practices without empirical validation.

Second, while the literature often emphasises ESG practices, environmental sustainability, CO₂ emissions, and renewable energy efforts in Malaysia, it tends to overlook the country’s persistent and substantial reliance on coal and natural gas for electricity production. This oversight is especially critical, given that the electricity sector is one of the largest contributors to national carbon emissions. According to the International Energy Agency (2025), in 2022, the electricity sector accounted for 48.8 percent of total carbon emissions, followed by the transport sector at 22.3 percent, the industrial sector at 15 percent, with the remainder attributed to other energy industries.

Therefore, this study addresses key research gaps in understanding the environmental impact of coal and gas consumption within Malaysia’s electricity sector. Despite ongoing efforts to adopt ESG practices, empirical evidence linking energy use to CO₂ emissions remains limited. Given Malaysia’s continued dependence on fossil fuels, this study explores how ESG practices can facilitate the transition to sustainable energy and help reduce carbon emissions. It aims to provide actionable insights for enhancing environmental sustainability within Malaysia’s electricity supply industry. The findings can guide policymakers in designing targeted regulations and incentives that promote ESG aligned energy practices, such as increasing renewable energy adoption, reducing reliance on coal and gas, and enhancing carbon reporting and accountability within the electricity sector. This can support Malaysia’s national goal of achieving net zero emissions by 2050.

However, this study also contributes to several theories throughout this investigation. This study is grounded in Stakeholder Theory [13] and Institutional Theory [14], which explain how external pressures and stakeholder expectations drive electricity supply companies in Malaysia to adopt ESG-aligned energy practices for environmental sustainability.

## 2. Literature review

Malaysia’s energy comprises a well-diversified array of sources that effectively balance the nation’s need for energy security, affordability, and sustainability. Energy consumption, however, generates certain adverse effects on the ecosystem and climate. Consequently, the sustainable utilisation of energy is receiving heightened focus in Malaysia. Malaysia possesses both conventional (non-renewable) and non-conventional (renewable) energy sources [15]. As a growing nation, Malaysia has experienced a continual increase in electricity demand over the previous two decades. Conventional fossil fuel energy sources, including oil, coal, and natural gas, mostly govern the electricity sector. Malaysia possesses significant renewable energy sources, primarily biomass and solar [16].

Energy consumption has increased due to Malaysia’s rapid economic expansion, potentially resulting in elevated CO2 emissions from the combustion of fossil fuel energy sources. Addressing this issue involves integrating clean energies by advocating for renewable energy sources such as solar power, biomass, wind energy, hydroelectricity, and others [17]. Future expansion in the energy sector will focus predominantly on emerging renewable energy sources. The transition to renewable energy can mitigate greenhouse gas (GHG) emissions, thus alleviating extreme weather and climate effects while providing a dependable, prompt, and economical energy supply [16].

### 2.1. Different energy uses in Malaysian electricity supply company

Malaysia’s electricity supply sector operates within a complex energy landscape, historically dominated by fossil fuels, primarily coal and natural gas, due to their cost effectiveness and reliability [18]. However, recent efforts have focused on diversifying the energy mix to include renewables such as hydroelectric, solar, wind, biomass, and biogas, aiming to enhance energy security and reduce environmental impacts [7, 19].

Fossil fuel consumption, especially coal, remains a major contributor to greenhouse gas emissions, posing significant environmental and health risks [19]. Although these fuels facilitate economic development, they accelerate climate change through global warming and air pollution. In contrast, renewable energy sources emit minimal greenhouse gases and support long term sustainability [20]. Malaysia’s commitments under the Paris Agreement and its Net Zero 2050 target further underscore the urgency of this transition.

Besides coal, gas, and renewables, oil continues to serve as a supplementary energy source, particularly in remote areas using gas turbines. To enhance system resilience, the sector is also exploring innovative solutions such as liquefied natural gas (LNG) imports and integrated gasification combined cycle (IGCC) technologies

Moreover, initiatives such as the adoption of smart grid technologies and demand side management by electricity providers like Tenaga Nasional Berhad (TNB) aim to optimise energy efficiency and reduce system losses [21]. These strategies, supported by policy incentives and growing public private partnerships, reflect Malaysia’s broader alignment with environmental, social, and governance (ESG) standards [22].

Correspondingly, national energy plans have been revised to reflect these ambitions, particularly in response to international climate commitments such as the COP26 Glasgow Pact, which called for the phasing down of coal and fossil fuel subsidies. In this context, Malaysia aims to increase the share of renewable energy to 31 percent of its electricity generation mix by 2025, as outlined in the 2022–2040 energy roadmap. This shift is particularly critical given Malaysia’s heavy reliance on coal and natural gas, both of which are finite and environmentally detrimental. Although the country possesses substantial natural gas reserves, it faces the risk of becoming a net importer due to rising demand and resource depletion. Therefore, the transition towards renewables is not just an environmental imperative but an economic necessity [23].

Despite these ambitions, the practical energy landscape paints a more concerning picture. According to the International Atomic Energy Agency (2025), carbon emissions from coal have risen dramatically, from 5.93 Mt in 2000 to 88.86 Mt in 2022, signalling that Malaysia’s dependence on coal remains a major barrier to decarbonisation. Carbon emissions from natural gas have fluctuated, but the overall emissions from natural gas are at a higher level (see Fig 1). This paradox highlights the gap between policy intent and implementation. While Southeast Asian nations are increasingly embracing renewables due to their geographic advantages, Malaysia’s emissions profile suggests that energy consumption is still a dominant force in power generation.

Given this scenario, scholars have increasingly recognised the importance of examining coal and natural gas consumption as proxies for overall energy usage in studies of carbon emissions. However, a critical gap remains: there is a noticeable lack of empirical research specifically investigating the relationship between energy consumption, particularly coal usage and natural gas, and carbon emissions within the context of Malaysian electricity supply companies. Furthermore, environmental governance, a core pillar of ESG, demands that companies not only comply with sustainability regulations but also actively pursue strategies to reduce carbon emissions. In this light, energy producers such as TNB bear a significant responsibility.

At present, TNB Genco holds the largest market share in Malaysia’s power generation sector, with a total contracted capacity of 13.76 GW [24]. Data suggest that TNB is one of the primary contributors to carbon emissions through its energy consumption patterns, especially in coal and natural gas – based power generation. Therefore, against this backdrop, it is imperative to conduct an empirical investigation on the impact of energy consumption on carbon emissions in the electricity production sector. Such research will not only fill a vital gap in the literature but also offer practical insights for improving environmental governance and advancing ESG performance among energy providers in Malaysia.

### 2.2. ESG practices in the electricity supply company of Malaysia

Malaysia’s electricity supply sector continues to depend heavily on fossil fuels, especially coal and natural gas, to meet growing energy needs. While these sources help ensure energy security, their environmental consequences are severe. The burning of fossil fuels releases large amounts of greenhouse gases, which contribute to climate change, air and water pollution, and the degradation of natural ecosystems). In addition, the processes of extracting and transporting these fuels often disrupt local environments and threaten biodiversity.

In response to these challenges, global and national policies have increasingly promoted the transition to cleaner and more sustainable energy sources. Malaysia’s participation in the Paris Agreement ([25] along with its [26] National Energy Policy (2022 reflects a commitment to reducing environmental damage and improving sustainability. The International Renewable Energy Agency (2023) further emphasises that sustainability should be considered throughout the entire energy lifecycle, from production to end use.

ESG practices go beyond environmental efforts and also address social responsibility and good governance. When implemented effectively, ESG strategies help companies improve operational efficiency, build stakeholder trust, and attract responsible investors [27]. Malaysia’s regulatory bodies, such as the Securities Commission and Bursa Malaysia, have introduced ESG guidelines that encourage companies to follow clear sustainability standards and enhance reporting practices [28].

Long term sustainability requires a shift in how companies operate and plan their energy use. This includes moving away from fossil fuels, increasing investment in renewable energy, and prioritising energy conservation. These actions are essential not only for reducing emissions but also for strengthening the resilience of the electricity supply sector [29]. Moreover, collaboration with stakeholders such as policymakers, civil society, and local communities ensures that ESG practices align with wider development goals [30].

Amid this evolving energy landscape, Malaysia’s main electricity provider, which owns nearly 51% of Malaysia’s power generation market share, Tenaga Nasional Berhad (TNB), has begun integrating Environmental, Social, and Governance (ESG) practices into its strategy. These include investments in renewable energy, setting emission reduction targets, and improving energy efficiency [31, 9]. In alignment with Malaysia’s net zero ambition, Tenaga Nasional Berhad (TNB) has committed to achieving net zero carbon emissions by the year 2050. This target forms part of TNB’s broader sustainability pathway, announced in 2021. As part of its immediate efforts, the company aims to reduce Scope 1 carbon emission intensity by 5 percent annually. TNB is confident in surpassing its interim target of a 35 percent reduction in carbon emission intensity by 2035, with a projected overall reduction of 46.47 percent by 2050.

To support this transition, TNB is working to secure up to 5GW of renewable energy (RE) capacity in Malaysia. This will be achieved through various national initiatives including the Large Scale Solar 5 (LSS5), the Corporate Renewable Energy Supply Scheme (CRESS), and the Corporate Green Power Programme (CGPP) [12]. Moreover, as part of its commitment to environmental stewardship, TNB launched the My Brighter Green Programme in conjunction with its 74th anniversary. Under this initiative, a total of 78,100 trees were planted across 42 designated areas nationwide, covering approximately 174 acres. This effort is estimated to have sequestered around 3,100 tonnes of CO₂ equivalent (tCO₂e), contributing meaningfully to natural carbon offsetting. Additionally, TNB introduced Project Dragon in June 2024 to explore the feasibility of carbon capture technologies. The project focuses on integrating Carbon Capture and Utilisation (CCU) technology at the 2000MW Jimah East Power (JEP) coal power plant. The pilot aims to capture up to 5,000 kg of CO₂ annually, with potential applications through biological or hydrogenation pathways. This represents a significant step toward technological innovation in emission reduction.

Together, these ESG initiatives reflect TNB’s holistic approach to sustainability, combining operational transformation, renewable energy expansion, and nature-based solutions. However, despite these efforts, the company remains heavily dependent on coal and natural gas for electricity generation (Figs 2 and 3). As such, the visibility and effectiveness of its ESG initiatives remain limited. A meaningful shift towards environmental sustainability requires a substantial reduction in reliance on fossil fuels. Achieving this goal depends on the development and implementation of robust policies that genuinely promote ESG integration within the energy sector.

Therefore, understanding the relationship between energy consumption and environmental sustainability is essential for evaluating the extent to which ESG principles are being upheld in Malaysia’s electricity sector. By examining how different energy sources, particularly the continued reliance on coal and gas versus the integration of renewable energy, affect environmental outcomes such as carbon emissions, we can assess whether ESG commitments are translating into measurable environmental benefits. This analysis also helps determine if corporate sustainability strategies align with national and global climate goals, and whether current energy practices support or hinder the sector’s progress toward achieving long term ESG targets. A deeper exploration of this relationship is crucial for supporting more effective policy design and guiding the country’s transition towards a cleaner, more sustainable, and environmentally responsible energy future. This study aims to contribute to this area by providing empirical insights into how energy use patterns impact environmental outcomes in the context of ESG practices.

### 2.3. Energy consumption and environmental sustainability nexus in Malaysia’s electricity supply sector

Environmental sustainability has become increasingly central to corporate agendas, particularly in Malaysia’s electricity supply sector. As the nation pursues economic development, balancing energy demands with environmental preservation has become a key priority. In this context, Environmental, Social, and Governance (ESG) practices offer a framework for embedding sustainability into corporate strategies and operations.

While there is growing recognition of ESG in the electricity sector, empirical research directly linking energy consumption to environmental sustainability remains limited. Existing studies tend to emphasise the potential of ESG practices without providing robust empirical validation. For example, Ismail et al. (2020) highlighted the role of ESG in managing environmental risks, reducing resource consumption, and building stakeholder trust. Similarly, [9] stressed the importance of transparency in ESG reporting, which allows companies to monitor environmental performance and implement corrective actions. [32] emphasised the value of multi-stakeholder engagement, noting that alignment with societal expectations is vital for inclusive and sustainable development.

Several studies have examined the electricity sector’s role in Malaysia’s carbon reduction efforts. [33] explored the impact of fuel mix changes on emissions and found that despite shifts towards hydro and coal, emissions remained high due to ongoing fossil fuel reliance. [34] pointed to slow decarbonisation in the power sector, driven by rising energy demand and high renewable energy costs. They argued that national energy policy has prioritised energy security over climate goals, and recommended greater efficiency, emissions trading, and adoption of international best practices to meet the 2030 carbon reduction target. [35] found a strong positive correlation between energy consumption and carbon emissions, driven by economic and population growth. Their study identified the electricity and transport sectors as major emission sources and recommended mechanisms like the Feed-in Tariff (FiT) to promote renewable energy and energy efficiency.

Moreover, [36] evaluated three alternative power generation scenarios using the LEAP model, demonstrating that renewable energy could meet future demand and reduce emissions. They called for policymakers to integrate such scenarios into national energy planning. Based on the existing plans and projections of Malaysia, the power sector is expected to increase its renewable energy capacity to 31% by 2025 and 40% in 2035, reducing the carbon intensity to GDP of the sector by 60% by as early as 2030, compared to the year 2000 baseline [37]. Despite these efforts, fossil fuels continue to dominate Malaysia’s electricity generation. As of 2022, about 80 percent of electricity came from coal (46.8 percent) and natural gas (International Energy Agency, 2025).

With growing electricity demand fuelled by economic expansion, the environmental consequences are intensifying. According to the Ministry of Energy, Science, Technology, Environment, and Climate Change (MESTECC), managing carbon emissions will be difficult without major energy efficiency reforms. The Twelfth Malaysia Plan (RMK 12) outlines a national commitment to net zero emissions by 2050. A joint study by WWF Malaysia, the Boston Consulting Group (BCG), and the government found Malaysia has been a net emitter since 2004, with energy sector decarbonisation identified as a key priority [38, 39].

However, despite policy-level initiatives, there remains a critical research gap regarding the role of individual electricity companies, particularly TNB, in either driving or mitigating emissions. Most existing studies focus on national trends and policy analysis, often overlooking company-level dynamics and the environmental impacts of energy consumption by major producers. Therefore, it is essential to examine how TNB’s energy consumption patterns influence environmental sustainability and whether alignment with ESG principles can meaningfully reduce emissions. This study seeks to fill this gap by investigating the environmental implications of energy use by TNB. It also aims to provide insights for policymakers and stakeholders to craft more effective ESG strategies, supporting Malaysia’s transition to a sustainable energy future.

The study hypothesizes that non renewable energy consumption, specifically coal and natural gas, negatively impacts environmental sustainability by increasing carbon emissions. This is supported by graphical evidence (Figs 2 and 3) showing a linear increase in emissions alongside rising non-renewable energy use, as well as literature from Sharif et al. [40] (2019)), Ali et al. [41] (2022), and [42] Ou et al. (2011) highlighting the adverse effects of energy consumption on sustainability. The low adoption of renewable energy in Malaysia limits its mitigating potential, emphasizing the need to focus on reducing non-renewable energy consumption to improve environmental sustainability.Therefore, the hypothesis is:

H_1_: Non renewable energy consumption reduces environmental sustainability

## 3. Methodology

### 3.1. Data and source

The time series data was collected from the report of the Tenaga Nasional Berhad (TNB) Sustainability Reports from 2016 to 2021 for all variables, since this company is one of the main market share holders in electricity production. At present, TNB Genco owns nearly 51% of Malaysia’s power generation market share with a total contracted capacity of 13.76GW [12]. The focused variables included environmental sustainability (CO2) = total direct and indirect GHG emissions Million tCO2e-yearly, COL = Coal consumption GJ (Gigajoule or GJ equals one billion joules) and GAS = Natural Gas consumption GJ. Besides, this study also considers some control variables, i.e., GCAP = Generating Capacity Total MW (Megawatts), and USER = Total customer accounts, to provide a comprehensive evaluation.

### 3.2. Empirical equations


$${CO2}_{i,t}=\beta_{1}+\beta_{2}{COL}_{i,t}+\beta_{3}{GAS}_{i,t}+\beta_{5}{GCAP}_{i,t}+\beta_{6}{USER}_{i,t}+\varepsilon_{i,t}$$ (1)

$${CO2}_{i,t}=\beta_{1}+\beta_{2}{COL}_{i,t}+\beta_{3}{GAS}_{i,t}+\beta_{5}{GCAP}_{i,t}+\beta_{6}{USER}_{i,t}$$ (2)

Where,

Environment Sustainability (CO2) = Total direct and indirect GHG emissions Million tCO2e -Yearly (the proxy variable of environmental sustainability to uphold ESG practice). COL = Coal consumption GJ, GAS = Natural Gas consumption GJ (Gigajoule or GJ equals one billion joules), GCAP = Generating Capacity Total MW (Megawatts), USER = Total customer accounts, and ε = error correction terms.

### 3.3. Econometric approach

This study employs a number of econometric approaches, i.e., the Prais-Winsten regression and the Ordinary Least Square (OLS) regression, for a number of reasons. Our data is a short time series dataset; therefore, we must use an approach which can produce strong estimations from the small dataset. [43] Beck and Katz (1996) argue that the Prais-Winsten regression approach is highly efficient in handling short time series data. Second, this method considers standard error terms in estimation. Third, if there are heteroscedasticity and autocorrelation issues in the data, this method is still able to avoid these disturbances [44]. It can also take into consideration any serial correlation that may exist in a time series dataset, although the process is more complicated. Transforming a data series to address first-order autoregressive (AR1) correlation problems and making the residual stationery, using a generalized linear square (GLS) regression function and producing more accurate and dependable forecasts. This method also provides “d” statistics, and the value ought to be between 0 and 4, where 0 denotes a complete negative correlation, 2 shows no autocorrelation, and a value going in the direction of 4 implies a positive correlation. This study also utilizes the OLS method to check the robustness.

## 4. Results and discussions

### 4.1. Descriptive statistics and correlation

The descriptive statistics presented in Table 1 offer an overview of the dataset’s key variables. The average carbon dioxide emissions (CO2) (the proxy variable of environmental sustainability to uphold ESG practice) are approximately 5.47, with a standard deviation of around 0.18. The range of CO2 values spans from a minimum of 5.27 to a maximum of 5.76. For the variable “coal,” the average is 8.59, with a standard deviation of about 0.06, ranging from 8.51 to 8.66. The variable “gas” has an average value of 8.25, a standard deviation of roughly 0.04, and values ranging from 8.21 to 8.31. In the case of the “user” variable, the average is 6.96, with a standard deviation of approximately 0.02 and values ranging from 6.93 to 6.99. Lastly, the variable “gcap” has an average value of 4.09, a standard deviation of about 0.05, and values ranging from 4.03 to 4.15. These statistics provide a summary of the central tendency and dispersion of each variable.

**Table 1:** Descriptive Statistics.

Variable	Observation	Mean	Std. dev.	Min	Max
CO2	6	5.474304	.1784411	5.27153	5.755194
COAL	6	8.588354	.0598064	8.513011	8.659883
GAS	6	8.25413	.035791	8.208941	8.310047
USER	6	6.956296	.0241812	6.926867	6.985131
GCAP	6	4.090123	.0519623	4.026922	4.147202

Table 2 shows a correlation matrix, which provides insights into the relationships between variables. For example, CO2 emissions (the proxy variable of environmental sustainability to uphold ESG practice) are positively correlated with coal and gas, negatively correlated with USER, and strongly negatively correlated with electric generation capacity. These correlations help understand how changes in one variable may be associated with changes in others.

**Table 2:** Correlation Matrix.

	CO2	COAL	GAS	USER	GCAP
CO2	1.0000				
COAL	0.6257	1.0000			
GAS	0.1572	0.2097	1.0000		
USER	−0.6698	0.8773	0.4739	1.0000	
GCAP	−0.6909	0.7710	0.5543	0.9800	1.0000

Source: author estimation.

Table 3 provides values of the multicollinearity test by the Variance Inflation Factor (VIF) test. The values of multicollinearity are mostly low to moderate. At the same time, the Table 2 a few of the independent variables show high correlations which often raise the issue of multicollinearity. However, before the VIF test, we transform the data to logarithmic form which actually occasionally helps in addressing moderate multicollinearity problems. And Table 4 shows mostly low multicollinearity which barely affects the reliability of the estimations of the study.

**Table 3:** Multicollinearity Test.

Variable	VIF
LUSER	10.74
LGCAP	8.38
LCOAL	3.11
LGAS	1.75

**Table 4:** Model Set Test for Prais–Winsten AR(1) Regression.

Source	SS	Df	MS	Number of obs	=	6
				F(4, 1)	>	99999.00
Model	9.251	4	2.312	Prob > F	=	0.00
Residual	4.704	1	4.704	R-squared	=	0.97
				Adj R-squared	=	0.96
Total	9.251	5	1.850	Root MSE	=	2.205

Source: Autho’s calculation.

### 4.2. Main results

Table 4 provides statistical information regarding the model fit of the Prais–Winsten AR(1) regression according to equation (1). The value of the F-statistic is very large (“> 99999.00”), indicating high statistical significance of the overall model. The value of R-squared is 0.97, which indicates a perfect fit, suggesting that the model explains all the variability in the dependent variable. The adjusted R-squared penalizes the model for including irrelevant predictors. Like R-squared, it is 0.96, indicating a perfect fit. In summary, the model appears to have a perfect fit (R-squared and Adj R-squared), and the F-statistic is highly significant, suggesting that the regression model is a good fit for the data.

Table 5 provides the results of the study from the Prais–Winsten AR(1) Regression. The coefficient of coal is 0.670, indicating that a one-unit increase in the use of coal as a raw material for electricity regeneration is associated with an increase of 0.670 units of CO2 emissions (the proxy variable of environmental sustainability to uphold ESG practice). The coefficient is highly significant at a 1% significance level. This indicates that the use of coal as a raw material by electricity production companies in Malaysia significantly contributes to CO2 emissions. Though Malaysia highly prioritizes environmental conservation and a low carbon emissions policy, the electricity production sector still has not yet implemented the low carbon emissions policy, especially coal use, due to time constraints. This result is highly in line with the number of prior findings [45, 46].

**Table 5:** Prais–Winsten AR(1) Regression with Iterated Estimates.

CO2	Coefficient	Std. err.	t	P > t	[95% conf.	interval
LCOAL	0.670***	0.001	594.45	0.001	0.684	0.655
LGAS	1.195***	0.0006	1738.13	0.000	1.186	1.204
LGCAP	−0.882***	0.002	1391.50	0.000	−0.918	−0.847
LUSER	1.266***	0.008	907.10	0.001	1.1643	1.367
cons	−37.107	0.038	−957.67	0.001	−37.599	−36.615
Durbin–Watson statistic (original) = 3.333, Durbin–Watson statistic (transformed) = 1.001						

Significance level 1%, 5% &10% denote by ***, ** & * respectively.

Table 5 also shows the role of gas used as the raw material in electricity production companies in Malaysia. The coefficient of gas use is 1.195, suggesting that a one-unit increase in the use of gas is associated with an increase of 1.195 units of CO2 emissions. Besides, the coefficient of gas use is strongly significant at a 1% significance level. This result is consistent with a number of previous studies [47]. These studies also report that the use of natural gas is also highly responsible for generating huge amounts of CO2, in addition to coal, to produce electricity. Though coal and natural gas are highly responsible for CO2 emissions in Malaysia, the amount of emissions is decreasing day by day, which we can find in Fig 1 due to the government’s promise to reduce the CO2 emissions.

At the same time, the government of Malaysia has also taken a number of initiatives to minimize the dependence on coal and gas to generate electricity. First, the government formulated low carbon emissions policies, Vision 2030, which will help the country minimize the use of minerals in electricity generation. Second, focus more on renewable energy sectors, i.e., solar, wind, and biomass, to diversify its energy mix and reduce dependence on fossil fuels. Following the new energy use policy, the electricity production sector of Malaysia is also diversifying their production. The electricity production sector also put ESG practices in place, and they have strong internal regulatory cells to maintain social and environmental responsibilities and contribute to sustainable development [48]. Third, the sector also promotes green technologies and green energy to reduce greenhouse gas emissions and uphold ESG procedures.

The table also provides the coefficients for the control variables. The coefficient of Gcap (electricity generation capacity) is −0.882, indicating that a one-unit increase in “Gcap” is associated with a decrease of 0.882 units of CO2 emissions. This is a very interesting result; if the current electricity demand rises and the sector increases the production but for the new production, the CO2 emissions will decrease instead of rising. This means the newly adopted low CO2 policies, renewable energy sources, and green technologies started to contribute to reducing CO2 emissions and strengthening ESG practices. However, some studies come up with the same findings [49].

Finally, the coefficient of User (number of users) is 1.266, suggesting that, holding other variables constant, a one-unit increase in the “User” is associated with an increase of 1.266 units of CO2 emissions. This is a bit alarming; if the use of electricity rises, the CO2 emissions also rise. It may be brought on by the recent intensive industrialization and the introduction of various internet-based devices. As a result, the industry that produces electricity must turn to gas and coal for further production because the amount of energy produced from renewable sources is insufficient to fulfil demand. While coal and gas still significantly contribute to CO2 emissions, this could be the reason for the significant increase of CO2 from one unit increase of User.

The Prais-Winsten AR(1) regression also provides the Durbin–Watson statistic along with the coefficients in Table 6. The Durbin–Watson statistic (original) is 3.333, and the transformed statistic is 1.001. These statistics detect the presence of autocorrelation in the residuals. A value close to 2 suggests no autocorrelation. In this case, the transformed statistic is very close to 2, indicating minimal autocorrelation. Overall, the model fits the data well, with statistically significant coefficients and a low Durbin–Watson statistic suggesting minimal autocorrelation in the residuals. This indicates that the method has the capacity to tackle the abnormal strength of the estimation.

**Table 6:** Results from Ordinary Least Squares (OLS).

CO2	Coefficient	Std. err.	t	P > t	[95% conf.	interval
LCOAL	0.0132*	0.0003	34.50	0.018	.018	.008
LGAS	0.003*	0.0002	13.96	0.046	.0003	.006
LGCAP	−1.43*	34.62	−36.23	0.018	−1.38	−1.48
LUSER	0.535*	0.132	34.27	0.019	0.853	1.217
cons	−2.070	634454.2	−32.55	0.020	−2.870	−1.260

Significance level 1%, 5% &10% denote by ***, ** & * respectively.

### 4.3. Robustness check with the Ordinary Least Square (OLS)

Table 6 presents the results from the OLS regression analysis followed by equation (2) as a robustness check. The coefficients represent that the use of coal and gas is associated with a significant increase in CO2 emissions. The results of both the variables from the OLS estimation are consistent with the results from the results of the Prais–Winsten regression presented in Table 4. At the same time, the electricity generation capacity (cap) and the number of users (user) exhibit significant negative and positive impacts on CO2 emissions, respectively, which is also harmonious with the results of Table 6.

## 5. Discussion

The study examines the impact of energy use, i.e., coal and gas as raw materials for electricity generation in Malaysia, on environmental sustainability using the proxy of CO2 emissions to uphold Environmental, Social, and Governance (ESG) practices in the electricity production sector of the county. As a stand-in for environmental sustainability in accordance with ESG principles, the coefficient estimates from the Prais–Winsten AR(1) Regression provide significant insights into the link between energy consumption and CO2 emissions (environmental sustainability).

The coefficients of gas and coal consumption are 1.195 and 0.670, respectively, which are strongly significant at the 1% significance level and highlight the important role that these energy sources play in environmental deterioration. Despite Malaysia’s dedication to low carbon emissions laws and environmental preservation, the country’s electricity production industry has still partially complied with these goals because of infrastructure installation constraints, particularly with regard to the use of coal and gas [50, 51]. This country is highly reliant on coal and gas energy to produce electricity, so any sudden policy change or adoption of new energy policies cannot easily replace the ongoing industrial setup [52]. Though the emissions have significantly decreased in recent years, they still lie at an alarming level. This progress implies that the electricity production sector strongly follows ESG practices but has yet to reach a tolerable level of CO2 emissions or environmental sustainability.

However, there are promising signs of progress in the electricity production sector. The Malaysian government has initiated several measures to reduce dependence on coal and gas for electricity generation, including the formulation of low carbon emissions policies and the promotion of renewable energy sources such as solar, wind, and biomass ([53] l., 2023). These efforts aim to diversify the energy mix, reduce greenhouse gas emissions, and uphold ESG principles. Additionally, the coefficients for control variables such as electricity generation capacity (Gcap) reveal a noteworthy trend: an increase in production capacity is associated with a decrease in CO2 emissions. This suggests that initiatives aimed at increasing energy efficiency and promoting green technologies are beginning to yield positive results in reducing CO2 emissions and strengthening ESG practices. Prior studies also highlight that although the Malaysian government has introduced initiatives to promote renewable energy, the country still heavily relies on fossil fuels for electricity generation, creating significant barriers to achieving sustainability in energy efficiency and the power sector. The slow pace of the renewable energy transition further deepens this dependency on fossil fuels, hindering efforts to shift towards cleaner energy sources.

However, challenges remain, as indicated by the coefficient estimate for the number of users (User), which shows a positive association with CO2 emissions. This underscores the need for continued efforts to balance energy demand with sustainable practices, particularly in the face of rapid industrialization and increasing energy consumption from various sources. Overall, these findings provide valuable insights for policymakers, industry stakeholders, and environmental advocates in crafting effective strategies to enhance environmental sustainability and uphold ESG principles in Malaysia’s energy sector.

In summary, the results from the Prais–Winsten AR(1) Regression and OLS regressions provide robust evidence of the relationship between energy consumption and CO2 emissions, contributing valuable insights to the discourse on environmental sustainability and ESG practices in the energy production sector of Malaysia.

## 6. Conclusion and policy implications

The aim of this study is to examine the effects of different energy consumption, i.e., coal and gas, on environmental sustainability using the proxy of CO2 emissions to uphold Environmental, Social, and Governance (ESG) practices in the electricity supply company (Tenaga Nasional Berhad) of Malaysia. We collected time series data from the sustainability reports of Tenaga Nasional Berhad (TNB), Malaysia’s sole electricity producer, spanning from 2016 to 2021, and employed Prais-Winsten regression and Ordinary Least Squares (OLS) regression methods to evaluate the impact.

The findings of the study reveal that coal and gas consumption exhibits a strong positive effect on CO2 emissions, indicating a significant role in contributing to environmental pollution and in further undermine environmental sustainability. However, the government’s initiatives, such as Vision 2030 and emphasis on renewable energy sources, are proving effective in reducing CO2 emissions despite an increase in electricity demand. Notably, the negative coefficient for Gcap suggests that increasing electricity production capacity leads to a decrease in CO2 emissions, highlighting the success of low CO2 policies and green technologies. Conversely, the positive coefficient for the number of users underlines the challenge posed by rising electricity consumption, potentially driven by industrialization, and technology adoption, necessitating a cautious approach to energy sourcing to mitigate environmental challenges. Moreover, the strict practice of ESG rules and regulations in the TNB also can contribute to uphold environmental sustainability.

Based on the findings of the study, we offer several policy recommendations for the electricity production sector of Malaysia, specifically targeting Tenaga Nasional Berhad, the country’s sole electricity producer. First, the electricity production sector of Malaysia (Tenaga Nasional Berhad) should diversify energy sources by promoting renewable energy, upgrading infrastructure, and implementing stricter emission standards, including carbon pricing mechanisms. Second, the industrial sector needs to focus on energy efficiency programs through mandatory energy audits and higher efficiency standards, supported by training programs and financial assistance for transitioning to cleaner technologies. Third, the residential and commercial sectors should manage energy demand with smart metering systems, offer energy efficiency rebates, and launch public awareness campaigns on energy conservation. Additionally, the transportation sector must promote electric vehicles by investing in charging infrastructure and expanding public transportation with green transit options. Finally, a comprehensive energy policy integrating efforts across all sectors, coupled with robust monitoring and evaluation frameworks, will ensure progress towards environmental sustainability and adherence to ESG principles in Malaysia.

Our study has some limitations even the study offers insightful information on the link between energy use and environmental sustainability channel through CO2 emissions. The study ignores other factors that may also affect environmental sustainability through ESG practice, such as technological improvements, renewable energy investments, energy efficiency measures, and socioeconomic variables. Second, the study only considered the Malaysian electricy producing company, while other sectors and the electricity supply companies from other countries are also disregarded. Finally, the study only focuses shrort span of period of the dataset to evaluate the factors.

The current study should be further enhanced by future research initiatives that include a wider variety of variables in order to thoroughly evaluate the factors that influence environmental sustainability and ESG practices in Malaysia’s energy industry. This could entail analyzing how institutional dynamics, consumer behaviour, technical innovation, and regulatory frameworks shape patterns of energy use and CO2 emissions. Moreover, longitudinal research that monitors the course of renewable energy policy implementation and its effects over time can offer important insights into how well policy interventions support sustainable energy transitions and help Malaysia meet its ESG goals and environmental sustainability in th electricity supply sector.
